# Human Endogenous Retrovirus-H-Derived miR-4454 Inhibits the Expression of *DNAJB4* and *SASH1* in Non-Muscle-Invasive Bladder Cancer

**DOI:** 10.3390/genes14071410

**Published:** 2023-07-07

**Authors:** Eun Gyung Park, Du Hyeong Lee, Woo Ryung Kim, Yun Ju Lee, Woo Hyeon Bae, Jung-min Kim, Hae Jin Shin, Hongseok Ha, Joo Mi Yi, Ssang Goo Cho, Yung Hyun Choi, Sun Hee Leem, Hee Jae Cha, Sang Woo Kim, Heui Soo Kim

**Affiliations:** 1 Department of Integrated Biological Sciences, Pusan National University, Busan 46241, Republic of Korea; ehdtodt@pusan.ac.kr (E.G.P.); doo2080@naver.com (D.H.L.); dnfud647@pusan.ac.kr (W.R.K.); lsg5821@naver.com (Y.J.L.); qodngus96@naver.com (W.H.B.); jmk95@naver.com (J.-m.K.); 0705haejin@naver.com (H.J.S.); 2 Institute of Systems Biology, Pusan National University, Busan 46241, Republic of Korea; 3 Division of Life Sciences, Korea University, Seoul 02841, Republic of Korea; hay2k@korea.ac.kr; 4 Department of Microbiology and Immunology, Inje University College of Medicine, Busan 47392, Republic of Korea; jmyi76@inje.ac.kr; 5 Department of Stem Cell & Regenerative Biotechnology, Institute of Advanced Regenerative Science, Konkuk University, Seoul 05029, Republic of Korea; ssangoo@konkuk.ac.kr; 6 Department of Biochemistry, College of Korean Medicine, Dong-Eui University, Busan 47227, Republic of Korea; choiyh@deu.ac.kr; 7 Department of Biological Science, Dong-A University, Busan 49315, Republic of Korea; shleem@dau.ac.kr; 8 Department of Parasitology and Genetics, College of Medicine, Kosin University, Busan 49104, Republic of Korea; hcha@kosin.ac.kr; 9 Department of Biological Sciences, College of Natural Sciences, Pusan National University, Busan 46241, Republic of Korea; kimsw@pusan.ac.kr

**Keywords:** human endogenous retrovirus, HERV-H-derived microRNAs, miR-4454, non-muscle-invasive bladder cancer, miRNAs derived from transposable elements, DnaJ heat shock protein family (Hsp40) member B4, SAM and SH3 domain containing 1

## Abstract

Although most human endogenous retroviruses (HERVs) have been silenced and lost their ability to translocate because of accumulated mutations during evolution, they still play important roles in human biology. Several studies have demonstrated that HERVs play pathological roles in numerous human diseases, especially cancer. A few studies have revealed that long non-coding RNAs that are transcribed from HERV sequences affect cancer progression. However, there is no study on microRNAs derived from HERVs related to cancer. In this study, we identified 29 microRNAs (miRNAs) derived from HERV sequences in the human genome. In particular, we discovered that miR-4454, which is HERV-H-derived miRNA, was upregulated in non-muscle-invasive bladder cancer (NMIBC) cells. To figure out the effects of upregulated miR-4454 in NMIBC, genes whose expression was downregulated in NMIBC, as well as tumor suppressor genes, were selected as putative target genes of miR-4454. The dual-luciferase assay was used to determine the negative relationship between miR-4454 and its target genes, *DNAJB4* and *SASH1*, and they were confirmed to be promising target genes of miR-4454. Taken together, this study suggests that the upregulation of miR-4454 derived from HERV-H in NMIBC reduces the expression of the tumor suppressor genes, *DNAJB4* and *SASH1*, to promote NMIBC progression.

## 1. Introduction

The human genome consists of approximately 50% repetitive DNA sequences, most of which are transposable elements (TEs) that can move into certain parts of the genome. Insertion of TEs can influence many DNA functions through epigenetic regulation, leading to the evolution of the human genome and causing various diseases [1,2,3,4,5]. TEs are largely classified into Class I retrotransposons and Class II DNA transposons based on their transposition mechanisms. Class I retrotransposons are generally transposed in the genome through a “copy-and-paste” mechanism. Based on the presence of long terminal repeat (LTR) elements at the flanking region, they are divided into LTR or non-LTR retrotransposons [6]. Most TEs have lost their transposition activity in the human genome, except for long-interspersed element 1 (LINE-1 or L1), which still plays important roles in gene function and genome evolution by regulating gene expressions directly or indirectly. According to recent studies, TEs contribute to the regulation of epigenetic gene expression by producing microRNAs (miRNAs) [2,7]. 

miRNAs are small non-coding RNA with sizes of 19–25 nucleotides that bind complementarily to the 3′ untranslated region (3′ UTR) of messenger RNA (mRNA), inhibiting translation and promoting degradation, thereby leading to gene silencing [8]. Typically, miRNAs are derived from intragenic or intergenic regions of the human genome and expressed with their host genes by the same promoter or are independently transcribed by their own promoters [9,10]. Recent studies have revealed that some of these miRNAs originate from TEs; these are called miRNAs derived from transposable elements (MDTEs). In our previous study, we investigated 474 MDTEs in the human genome using the latest versions of genomic databases and assessed the relationship between MDTEs and human diseases. However, studies on miRNAs derived from human endogenous retroviruses (HERVs) are lacking. In the present study, we narrowed down the types of miRNAs derived from HERVs and attempted to reveal the correlation between HERV-derived miRNAs and cancer.

HERVs are a subfamily of LTR constituting approximately 8% of the human genome. They were acquired from the human genome through infection with exogenous retroviruses during primate evolution [11,12]. Most HERVs invaded the primate genome during the early radiation of primates about 50–10 million years ago and were inherited similarly to other cellular genes [13]. Full-length HERVs have the viral genetic structures of gag, pro, pol, and env as internal regions and two LTR structures at each end [11,14]. HERV elements are classified into three groups: Class I (Genus Gammaretrovirus) with HERV-F, H, I, E, R, P, T, W; Class II (Genus Betaretrovirus) with HERV-K; and Class III (Genus Spumavirus-related) with HERV-L [15,16]. The HERV-H family, which is the most abundant family in the human genome, is considered one of the most important targets for research on biological functions. HERV-H was inserted into the primate genome 40 million years ago and is flanked by the LTR7 family [17,18]. This family is further subdivided into four subfamilies based on distinct LTR consensus sequences: LTR7 (formerly known as Type I), 7b (Type II), 7c, and 7y (Type Ia) [19]. Regarding the biological functions of HERV-H, one study revealed that extremely high expression of HERV-H RNA in human embryonic stem cells (hESCs) contributes to pluripotency by providing binding sites for pluripotency transcription factors and can therefore be used as a marker for hESCs [20,21]. HERV-H also creates topologically associating domain (TAD) boundaries in human pluripotent stem cells (hPSCs) and regulates the transcription of upstream genes [22]. Furthermore, it is also known that HERV-H activity is important for induced pluripotent stem cells (iPSCs) generation. A previous study demonstrated that during iPSC reprogramming, KLF4 facilitates the binding of Yamanaka factors, including Oct3/4 and SOX2, to LTR7. Consequently, HERV-H undergoes temporary hyperactivation, promoting the formation of iPSCs [23,24]. These previous studies indicate that HERV-H plays a crucial role in maintaining cellular pluripotency in stem cells. HERV-H expression is also associated with human diseases, particularly cancer progression. It was revealed that HERV-H is involved in immunosuppression by helping cancer cells evade the immune system [25,26]. For instance, the expression of HERV-H tends to increase in cancers such as colorectal cancer, gastric cancer, and prostate cancer, and in many cancer cell lines [18,27,28,29].

In this study, we identified 29 miRNAs derived from HERVs. Among these, miR-4454 originated from HERV-H, which is closely associated with human diseases [30]. Previous studies have shown that miR-4454 is differentially expressed in various types of cancers and acts in different ways. Upregulated miR-4454 shows oncogenic traits in hepatocellular carcinoma and cervical cancer and promotes cancer cell proliferation, invasion, and migration [31,32]. In contrast, in colorectal cancer and ovarian cancer, downregulation of miR-4454 induces cancer progression [33,34]. In the case of bladder cancer, a previous study revealed the expression profiles of miRNAs in non-muscle-invasive bladder cancer (NMIBC) patient samples [35]. From the study, the expression of miR-4454 was increased in three types of NMIBC samples: tumors, urine exosomes, and white blood cells (WBCs). However, to the best of our knowledge, there have been no further studies on the mechanism of gene regulation by miR-4454 in NMIBC. Therefore, in this study, we investigated whether upregulated miR-4454 derived from HERV-H has oncogenic traits by targeting tumor suppressor genes in NMIBC.

## 2. Materials and Methods

### 2.1. Identification of miRNAs Derived from HERVs

HERV-derived miRNAs were identified as described in our previous study [30]. Briefly, to investigate the coordinates of MDTEs in the human genome, the chromosomal locations of mature miRNAs were obtained from the miRBase v22 database and intersected with repeat sequences downloaded from the UCSC table browser (GRCh38/hg38) using the IntersectBed module from Bedtools. Based on these results, we selected only miRNAs that overlapped with the internal portion or associated LTRs of the HERV family. Dot plot analysis was performed using the D-GENIES database (https://dgenies.toulouse.inra.fr/ (accessed on 20 April 2023)) to align the HERV-H int sequence, from which the miRNA was derived, to the HERV-H consensus sequence. The HERV sequences were downloaded from the HERVd database (https://herv.img.cas.cz/ (accessed on 13 April 2023)).

### 2.2. Cell Culture

All cells were cultured at 37 °C in a 5% CO_2_ incubator in cell culture medium containing 10% heat-inactivated fetal bovine serum (FBS) (Gibco, Grand Island, NY, USA) and 1% penicillin/streptomycin (WELGENE, Gyeongsan, Republic of Korea). The normal human urothelial cell line, SV-HUC-1, was cultured in Ham’s F-12K (Kaighn’s modification) medium (Gibco, NY, USA). The NMIBC cell line, RT-4, and the human embryonic kidney-derived cell line, HEK293A, were cultured in Dulbecco’s modified Eagle’s medium (DMEM) (HyClone™, Logan, UT, USA).

### 2.3. RNA Extraction and Complementary DNA Synthesis

Total RNA was isolated from cells using Hybrid-R^TM^ (GeneAll, Seoul, Republic of Korea) according to the manufacturer’s instructions. The HB miR Multi Assay Kit^TM^ (SYSTEM I; HeimBiotek, Seoul, Republic of Korea) and PrimeScript^TM^ RT Reagent Kit with gDNA Eraser (TaKaRa, Shiga, Japan) were used for the reverse transcription of miRNA and mRNA, respectively, in accordance with the manufacturer’s recommendations. The thermal cycler (Eppendorf, Hamburg, Germany) was used for complementary DNA (cDNA) synthesis. The cycling conditions were as follows: HB miR Multi Assay Kit^TM^ (System I): incubation at 37 °C for 60 min, followed by incubation at 95 °C for 5 min, and then holding at 4 °C; PrimeScript^TM^ RT reagent Kit with gDNA Eraser: incubation at 37 °C for 15 min, followed by incubation at 85 °C for 5 s, and then holding at 4 °C. Each of the final cDNA samples was stored at −20 °C.

### 2.4. Quantitative Real-Time Polymerase Chain Reaction (qRT-PCR)

Expression levels of miRNA and their target genes were assessed by performing qRT-PCR in a Quantstudio1 system (Applied Biosystems, Foster City, CA, USA) using 10 ng of each cDNA sample. The expression of miR-4454 was measured using the HB_I Real-Time PCR Master Mix Kit (HeimBiotek, Seoul, Republic of Korea), and the small nuclear RNA (snRNA) U6 was used as a reference gene. The conditions were as follows: hold at 95 °C for 15 min for initial activation, followed by 40 thermal cycles at 95 °C for 10 s and 60 °C for 40 s; standard melting conditions for 90 s at 55 °C, and then for 5 s each at 1 °C increments between 55 °C and 99 °C. To examine the expression of the target genes, DnaJ heat shock protein family (Hsp40) member B4 (*DNAJB4*) and SAM and SH3 domain containing 1 (*SASH1*), SYBR Green Q-PCR Master Mix with Low Rox (SmartGene, Daejeon, Republic of Korea) was used. Glyceraldehyde 3-phosphate dehydrogenase (*GAPDH*) was used as the reference gene. Gene-specific primers designed using Primer3 v. 4.1 (https://primer3.ut.ee/ (accessed on 22 April 2023)) are listed in Table 1. The conditions were as follows: hold at 95 °C for 2 min for polymerase activation, followed by 45 thermal cycles at 95 °C for 5 s, 56 °C for *DNAJB4* and 60 °C for *SASH1* for 30 s, and 72 °C for 30 s; standard melting conditions for 15 s at 95 °C, 60 °C for 1 min followed by 95 °C for 1 s. The ramp rate of the last transformation from 60 °C to 95 °C was set at 0.15 °C/s. All samples were analyzed in triplicate, and the relative expression data were analyzed using the 2^−ΔΔCt^ method.

### 2.5. Identification of the Putative Tumor Suppressor Targets of miR-4454 in Bladder Cancer Cells

Tumor suppressor genes specifically regulated by miR-4454 in BC cells were identified using web-based databases and publicly available gene expression data. The TargetScanHuman database (release 7.2) was used to predict the putative targets of miR-4454. In addition, the list of tumor suppressor genes that were downregulated in bladder urothelial carcinoma samples was obtained from the tumor suppressor gene database TSGene 2.0 (https://bioinfo.uth.edu/TSGene (accessed on 21 April 2023)). Downregulated genes in NMIBC samples were analyzed using data from 16 NMIBC patients with no intravesical therapy in stage TaN0M0 compared to 9 normal controls obtained from patients with benign disease in the Gene Expression Omnibus (GEO) (accession number: GSE13507). Common genes that met all three conditions were identified using Venny 2.1.0 (https://bioinfogp.cnb.csic.es/tools/venny/ (accessed on 21 April 2023)). The secondary structure and minimum free energy (MFE) of the hybridization of miR-4454 and its target genes were predicted using the Bielefeld University Bioinformatics Server (BiBiServ) RNA Hybrid (https://bibiserv.cebitec.uni-bielefeld.de/rnahybrid (accessed on 25 April 2023)).

### 2.6. Dual-Luciferase Reporter Assay

To verify the interactions between miRNA and its target genes, dual-luciferase assays were conducted. The 3′ UTR of *DNAJB4* or *SASH1* containing the putative binding site of miR-4454 was cloned into a dual-luciferase vector, psi-CHECK2 (Promega, Madison, WI, USA). HEK293A cells were plated in 24-well plates at a density of 2.5 × 10^5^ cells/well. After 24 h, 50 nM of miR-4454 mimic/inhibitor or negative control and 500 ng each of *DNAJB4*-3′UTR vector/*SASH1*-3′UTR vector were co-transfected into cells using the JetPRIME reagent (Polyplus, Illkirch, France). The luciferase activities of the firefly and *Renilla* were measured 24 h after co-transfection by a Glomax^®^ 20/20 Luminometer (Promega, Madison, WI, USA), using a dual-luciferase reporter system (Promega, Madison, WI, USA) following the manufacturer’s suggestions. The activity of *Renilla* luciferase was used as an internal control for firefly luciferase measurements.

### 2.7. Statistical Analyses

Each experiment was conducted at least three times or in triplicate, and the mean ± standard deviation (SD) of the data was plotted on a bar graph. Differences among multiple groups were compared using one-way ANOVA, or differences between two groups were compared using Student’s *t*-test. Statistical significance was defined as *p* < 0.05.

## 3. Results

### 3.1. Identification of miRNAs Derived from HERVs in the Human Genome

In our previous study, we identified 55 MDTEs generated by an LTR retrotransposon [30]. In this study, among the 55 LTR-derived miRNAs, we considered not only the TE subclasses but also the families from which the miRNAs originated. To identify miRNAs derived from HERVs, we excluded miRNAs that originated from the ERVL-MaLR or gypsy families. As a result, there were 29 miRNAs derived from HERV sequences in the human genome, consisting of 19 miRNAs from ERV1, 9 miRNAs from ERVL, and only 1 miRNA from ERVK (Table 2).

**Figure 1:**
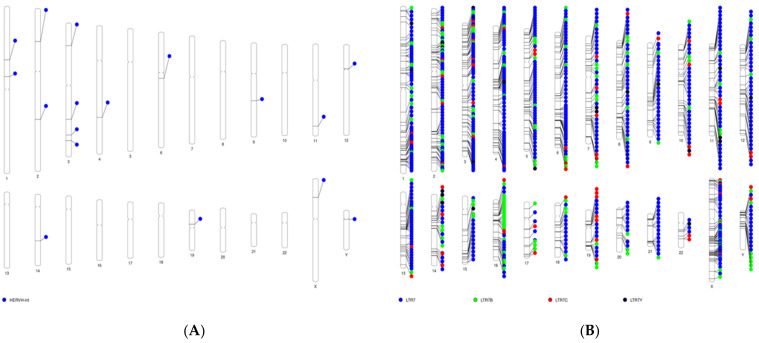
Distribution of the (**A**) full-length HERV-H family and the (**B**) solitary LTR7 families in the human genome.

Of these miRNAs, there were three HERV-H-derived miRNAs, the aim of this study; two miRNAs (miR-4454 and miR-7975) originating from the HERV-H-int element; and miR-6839-5p, originating from LTR7C. To identify whether the HERV-H elements from which these miRNAs were derived were from the full-length HERV-H or solitary LTR7 families, we confirmed the chromosomal locations of full-length HERV-H and solitary LTRs in the human genome. The strategy used to determine the coordinates of the full-length HERV-H or solitary LTR7 families is shown in Supplementary Figure S1. Considering the size of the consensus sequences of the internal region of HERV-H, we selected a full-length element with an internal region of >7 kb and two flanking LTRs. For the selection of solitary LTR7 families, the size of the LTR7 families was narrowed down to those larger than 435 and smaller than 465, taking into account internal deletions of approximately 15 bp. Among them, LTRs without HERV-H int within ±200 bp were considered to be a solitary LTR7 families. Consequently, 17 full-length HERV-H elements and 931 solitary LTR7 families were identified in the human genome (Figure 1A,B). The 931 solitary LTR7 families included 680 LTR7, 155 LTR7B, 69 LTRC, and 27 LTRY.

### 3.2. Sequence Conservation of HERV-H-int-Derived miRNAs

**Figure 2:**
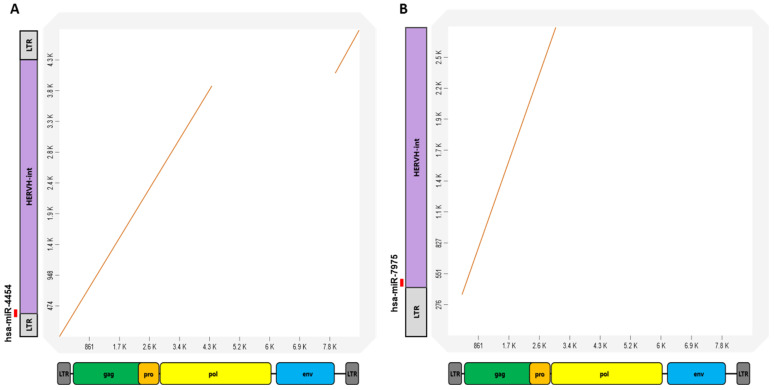
Dot plot illustrates the pairwise alignment of the HERV-H element that has been identified as the source of miRNAs against the consensus sequence of HERV-H. (**A**) The result of pairwise alignment indicates that HERV-H, which produces miR-4454, is conserved up to the middle of the pol region of the HERV-H consensus sequence. (**B**) Pairwise alignment results of HERV-H that produced miR-7975 showed that it has been conserved up to the pro region of the HERV-H consensus sequence.

Dot plot analysis was used to compare the HERV-H int element containing the region for miR-4454 or miR-7975 transcription with the consensus sequences of full-length HERV-H. The results of the pairwise alignment indicated that the internal region of HERV-H, which produces miR-4454, is conserved up to the middle of the pol region of the HERV-H consensus sequence, and for miR-7975, it is conserved up to the pro region of the HERV-H consensus sequence. Therefore, these two miRNAs did not originate from the full-length HERV-H (Figure 2A,B). The LTR7C sequences from which miR-6839-5p originated were not considered to belong to a solitary LTR7 family, as their genome size of 63 bp was much smaller than the consensus sequences of LTR7C.

### 3.3. Expression of miR-4454 in Bladder Cancer Cell Lines

**Figure 3:**
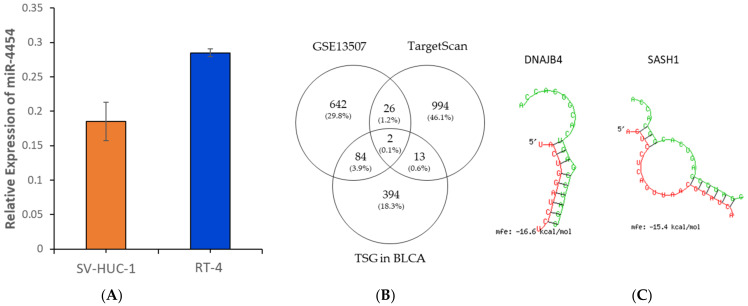
Relative expressions of (**A**) miR-4454 in SV-HUC-1 and RT-4 cells. (**B**) Venn diagram showing the number of common target genes for miR-4454. (**C**) The 3′ UTR sequences of target genes, *DNAJB4* and *SASH1*, and miR-4454 (green) were hybridized using the RNAhybrid program. The black box and the minimum free energy (MFE) value of the binding sequence is −16.6 kcal/mol and −15.4 kcal/mol, respectively.

As a previous study only showed significant upregulation of miR-4454 in three types of bio-specimens (tumors, white blood cells, and urine exosomes) of NMIBC patients [35], we compared the expression of miR-4454 using the NMIBC cell line, RT-4, and the normal human urothelial cell line, SV-HUC-1. The results showed that miR-4454 expression was significantly upregulated in RT-4 cells compared to SV-HUC-1 cells, consistent with a previous study (Figure 3A).

### 3.4. DNAJB4 and SASH1 Are Putative Tumor Suppressor Target Genes of miR-4454 in Bladder Cancer

Because the expression of miR-4454 was upregulated in RT-4, an NMIBC cell line, we hypothesized that miR-4454 tends to have oncogenic activity. Based on these assumptions, we aimed to identify the target genes of miR-4454, which are tumor suppressor genes with reduced expression in NMIBC. First, we identified the putative targets of miR-4454 using TargetScanHuman 7.2, and the number of predicted target genes were 1035. Next, we searched for differentially expressed genes in NMIBC patients using a publicly available gene expression dataset from the Gene Expression Omnibus (GEO) (accession number: GSE13507). Among these, 754 genes that were downregulated (log2Foldchange < −1.1) in patients with NMIBC were selected for analysis. Finally, we identified 493 tumor suppressor genes that were downregulated in bladder urothelial carcinoma samples compared to normal tissue samples from TCGA using the tumor suppressor gene database, TSGene 2.0 (https://bioinfo.uth.edu/TSGene (accessed on 21 April 2023)). There were only two common genes that satisfied all three conditions, *DNAJB4* and *SASH1* (Figure 3B). The structural interaction between miR-4454 and the 3′ UTR of *DNAJB4* or *SASH1*, generated by the BiBiServ RNA hybrid database, is shown in Figure 3C. The minimum free energy (MFE) value between miR-4454 and *DNAJB4* is −16.6 kcal/mol, and that between miR-4454 and *SASH1* is −15.4 kcal/mol.

### 3.5. The Negative Relationship between miR-4454 and DNAJB4/SASH1

**Figure 4:**
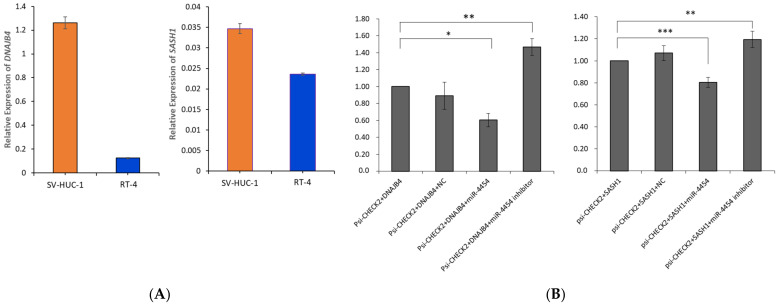
Relative expressions of *DNAJB4* and *SASH1* and dual-luciferase reporter assay. (**A**) Relative expressions of *DNAJB4* and *SASH1* were examined by qRT-PCR in SV-HUC-1 and RT-4 cells. (**B**) The interactions between miR-4454 and *DNAJB4* or *SASH1* were confirmed by the dual-luciferase reporter assay. HEK293A cells were co-transfected with psi-CHECK2 vectors containing the miR-4454 binding site of *DNAJB4* or *SASH1*, and the miR-4454 mimic/inhibitor or negative control. * *p* < 0.05, ** *p* < 0.01, *** *p* < 0.001 compared with control.

The relative expression levels of *DNAJB4* and *SASH1* were measured in SV-HUC-1 and RT-4 cells by qRT-PCR. In contrast to miR-4454, the levels of *DNAJB4* and *SASH1* expression were significantly lower in RT-4 cells than in SV-HUC-1 cells, supporting the results of our analysis (Figure 4A). To verify whether these were the meaningful target genes of miR-4454, a dual-luciferase reporter assay was conducted. The dual-luciferase vector, psi-CHECK2, that contains the putative binding sequences in the 3′ UTR of *DNAJB4* or *SASH1* was prepared for the experiment. The results showed that in HEK293A cells co-transfected with the constructed vector and the miR-4454 mimic, firefly luciferase activity was significantly inhibited, but not with the negative control, whereas the inhibition of miR-4454 revealed the opposite result (Figure 4B).

## 4. Discussion

Several studies have revealed that a large number of miRNAs are derived from TEs, accounting for about 15% of the total human miRNAs [30,36,37,38]. These MDTEs are evolutionarily less conserved than other miRNAs and are associated with various human diseases [36,39]. Similar to other TEs, HERVs can be transcribed into non-coding RNAs (ncRNAs) that act as regulatory elements that modulate gene expression and play pathological roles in numerous human diseases, especially cancer [40]. Several studies have focused on HERV-derived long ncRNAs in cancer [41,42]; however, few have included HERV-derived miRNAs. In our previous study, we investigated disease-related MDTEs and emphasized the importance of studying the unknown functions of these MDTEs to provide a deeper understanding of disease pathogenesis. In the present study, we focused on HERV-H-derived miRNAs and attempted to determine their association with cancer. By examining the overlapping regions between the coordinates of the HERV sequences and the miRNA gene locations, we identified 29 miRNAs derived from HERVs and 3 miRNAs (miR-4454, miR-7975, and miR-6849-5p) derived from HERV-H. To verify the relationship between these miRNAs and cancer, we first investigated the previously reported studies on these three miRNAs. One study revealed that miR-7975 does not form the hairpin structure of the precursor miRNA and presumed that miR-7975 may not be a true miRNA [43]. For miR-6849-5p, we could not find any studies related to cancer. On the other hand, miR-4454 has been shown to affect various types of cancers, including hepatocellular carcinoma (HCC), cervical cancer, ovarian cancer, and colorectal cancer, and shows the opposite action in each cancer type [31,32,33,34]. For instance, inhibition of miR-4454 in HepG2 cells induced Vps4A and Rab27A expressions and suppressed the proliferation, migration, and apoptosis of HepG2 cells to inhibit HCC progression [31]. In contrast, in colorectal cancer, the overexpression of miR-4454 reduced the survival of cancer cells dependent on GNL3L/NF-kB signal, supporting its role as a tumor suppressor [33]. Furthermore, there was a study related to bladder cancer with miR-4454. In an expression profiling study of differentially expressed miRNAs in NMIBC patient samples, miR-4454 was upregulated in three bio-specimen sources (white blood cells, tumors, and urine exosomes) [35]. However, the mechanism by which miR-4454 affects the onset and progression of cancer in NMIBC has not been reported and still remains unknown. Because the expression of miR-4454 is upregulated in NMIBC, we assumed that it would be oncogenic. Therefore, through bioinformatics analysis using the GEO dataset, TargetScanHuman, and TSGene 2.0 database, we selected a set of tumor suppressor genes that were reduced in NMIBC as putative targets of miR-4454. Two target genes, *SASH1* and *DNAJB*, satisfied each condition.

SAM and SH3 domain containing 1 (*SASH1*) is a signal adapter protein of the SLY family, a well-known tumor suppressor gene that shows decreased expression in various cancers such as breast cancer, colorectal cancer, non-small-cell lung cancer (NSCLC), gastric cancer, and cervical cancer [44,45,46,47,48,49]. Overexpression of *SASH1* inhibited proliferation and invasion in a human lung cancer cell line and suppressed TGF-β1-mediated epithelial-mesenchymal transition (EMT), migration, and invasion in a human gastric cancer cell line [50,51]. DnaJ heat shock protein family (Hsp40) member B4 (*DNAJB4*), also called *HLJ1*, is another well-known tumor suppressor gene that inhibits the invasion, migration, and proliferation of cancer cells [52]. It has been reported that *DNAJB4* expression generally tends to decrease in various cancers, such as colorectal cancer, breast cancer, and lung cancer [53,54,55]. One study showed that overexpression of *DNAJB4* indirectly upregulated the expression of E-cadherin or regulated the complex formation of NPM1 and inhibited cell invasion and migration in highly invasive lung cancer [55]. Therefore, overexpression of *DNAJB4* can suppress cancer progression and invasion and is considered a cancer biomarker or a promising target for anticancer therapy.

Taken together, these results suggest that the downregulation of these two genes through the upregulation of miR-4454 may promote NMIBC progression. To verify whether miR-4454 bound to these two genes and inhibited their expression, we conducted a dual-luciferase assay. As a result, we found that the luciferase activity was significantly reduced when miR-4454 was overexpressed or increased inversely in the presence of the specific inhibitor of miR-4454. Based on these results, we identified a negative correlation between miR-4454 and *DNAJB4*/*SASH1*, indicating that these two genes are the true target genes of miR-4454. Moreover, during the experiment, we identified the expression of miR-4454 in muscle-invasive bladder cancer (MIBC) cell lines, including 5637, T24, J82, HT-1376, and UMUC-3 (Supplementary Figure S2). As a result, the expression of miR-4454 was significantly upregulated only in the RT-4 cells, but not in the MIBC cells. Therefore, miR-4454 may also serve as a specific biomarker for the diagnosis of NMIBC, an early stage of bladder cancer. Although further studies should be conducted to discern the mechanisms of NMIBC progression, this study is significant for identifying practical target genes of miR-4454 and for investigating and organizing HERV-derived miRNAs for the first time.

## Figures and Tables

**Table 1:** Primer sequences used in this study.

Gene	Forward Primer (5′ to 3′)	Reverse Primer (5′ to 3′)
*SASH1*	GAATCCTGACCATGTCCATCCC	AATATGCAGCCTCACAGTGACC
*DNAJB4*	AGTGAGAATGATGTTTTGAAGCA	AGGGTAAACTTTATGGCATGTAA
*GAPDH*	GAAATCCCATCACCATCTTCCAGG	GAGCCCCAGCCTTCTCCATG

**Table 2:** Identification of miRNAs derived from HERVs in the human genome.

Chr	Start	End	miRNA	Family	Subfamily
chr1	51,059,879	51,059,900	hsa-miR-4421	ERV1	MER52A
chr1	51,060,031	51,060,054	hsa-miR-6500-5p	ERV1	MER52A
chr1	51,060,072	51,060,092	hsa-miR-6500-3p	ERV1	MER52A
chr1	237,471,164	237,471,185	hsa-miR-4428	ERV1	LTR9A1
chr3	32,506,342	32,506,362	hsa-miR-548ay-5p	ERV1	LTR43
chr3	32,506,305	32,506,326	hsa-miR-548ay-3p	ERV1	LTR43
chr3	164,171,523	164,171,544	hsa-miR-1263	ERV1	MER39
chr3	176,515,103	176,515,120	hsa-miR-7977	ERV1	HUERS-P3-int
chr4	163,093,607	163,093,626	hsa-miR-4454	ERV1	HERV-H-int
chr6	155,946,809	155,946,829	hsa-miR-1202	ERV1	MER52A
chr7	64,679,069	64,679,090	hsa-miR-6839-5p	ERV1	LTR7C
chr8	1,801,133	1,801,154	hsa-miR-3674	ERV1	LTR8A
chr12	122,695,964	122,695,985	hsa-miR-9902	ERV1	HUERS-P1-int
chr15	85,825,655	85,825,673	hsa-miR-548ap-5p	ERV1	MER50
chr15	85,825,692	85,825,710	hsa-miR-548ap-3p	ERV1	MER50
chr17	82,668,281	82,668,301	hsa-miR-4525	ERV1	MER52D
chr18	39,622,153	39,622,172	hsa-miR-924	ERV1	MER101B
chr19	12,920,373	12,920,394	hsa-miR-5695	ERV1	MER39B
chr19	55,123,225	55,123,242	hsa-miR-7975	ERV1	HERV-H-int
chr3	183,886,860	183,886,879	hsa-miR-4448	ERVK	LTR13
chr2	12,199,138	12,199,159	hsa-miR-3681-5p	ERVL	LTR16D1
chr2	12,199,174	12,199,195	hsa-miR-3681-3p	ERVL	LTR16D1
chr4	66,276,890	66,276,911	hsa-miR-1269a	ERVL	MLT2B3
chr8	28,505,126	28,505,142	hsa-miR-4288	ERVL	LTR86B1
chr14	67,441,902	67,441,922	hsa-miR-5694	ERVL	MLT2B1
chr16	14,907,756	14,907,779	hsa-miR-3670	ERVL	LTR16A1
chr17	12,917,313	12,917,334	hsa-miR-1269b	ERVL	MLT2B3
chr20	60,308,534	60,308,552	hsa-miR-646	ERVL	LTR67B
chr22	30,731,569	30,731,590	hsa-miR-3200-5p	ERVL	LTR16D2

## Data Availability

A publicly available dataset was analyzed in this study. The results shown in this study are based upon data from the Gene Expression Omnibus (GEO) database (accession number: GSE13507).

## Supplementary Materials

The following supporting information can be downloaded at: https://www.mdpi.com/article/10.3390/genes14071410/s1, Figure S1: Summary of the workflow.; Figure S2: Relative expression of hsa-miR-4454 in bladder cancer cell lines.
