# Supplementary material for: Human Endogenous Retrovirus-H-Derived miR-4454 Inhibits the Expression of DNAJB4 and SASH1 in Non-Muscle-Invasive Bladder Cancer
Source: Genes (Basel). 2023 Jul 7;14(7):1410. doi: 10.3390/genes14071410 (PMC10379226; doi:10.3390/genes14071410)
Supplement: Supplementary file 1 [file genes-14-01410-s001.zip › genes-2477498-supplementary.pptx]

## Slide 1
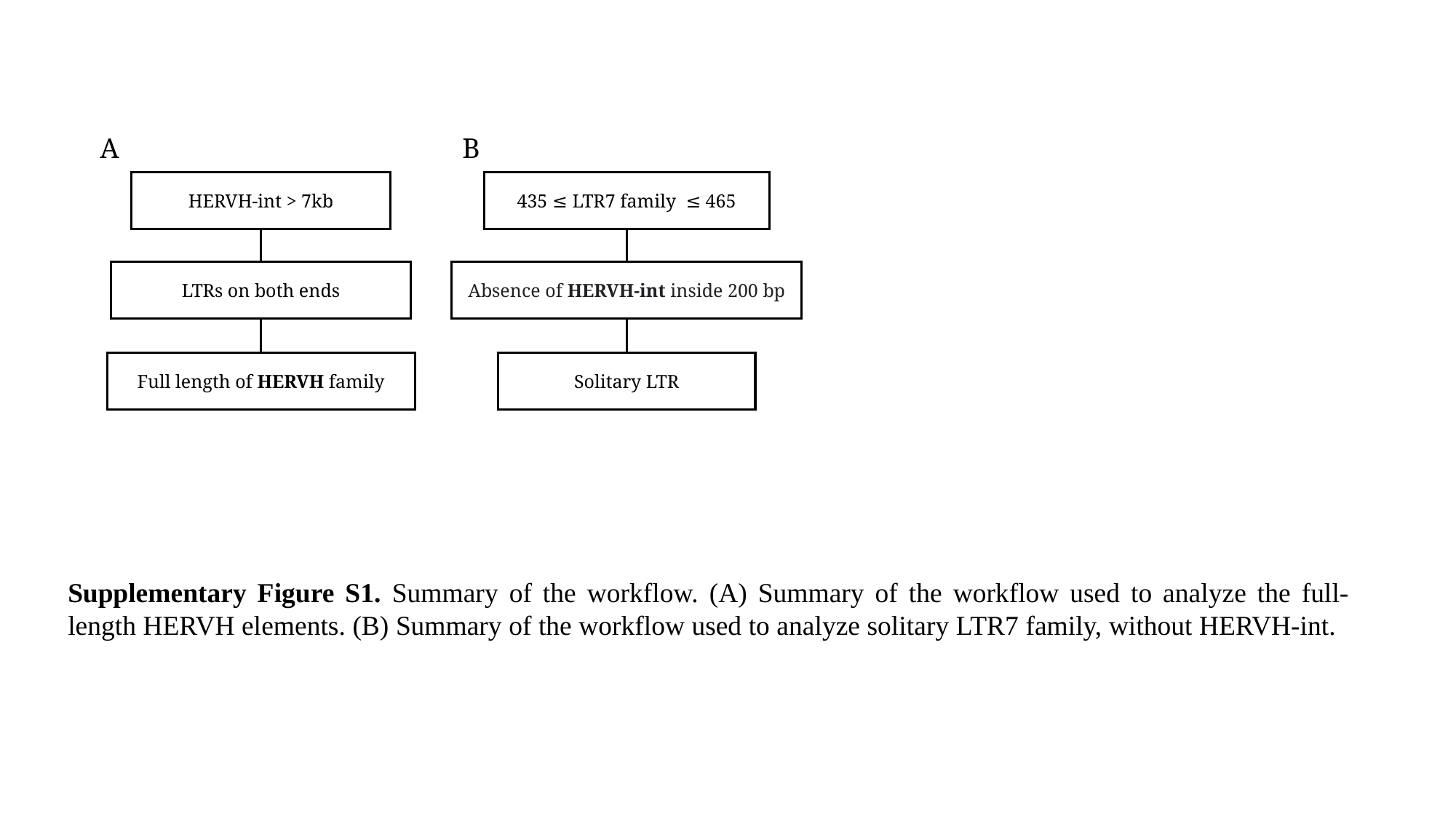

A
B
HERVH-int > 7kb
435 ≤ LTR7 family ≤ 465
LTRs on both ends
Absence of HERVH-int inside 200 bp
Full length of HERVH family
Solitary LTR
Supplementary Figure S1. Summary of the workflow. (A) Summary of the workflow used to analyze the full-length HERVH elements. (B) Summary of the workflow used to analyze solitary LTR7 family, without HERVH-int.

## Slide 2
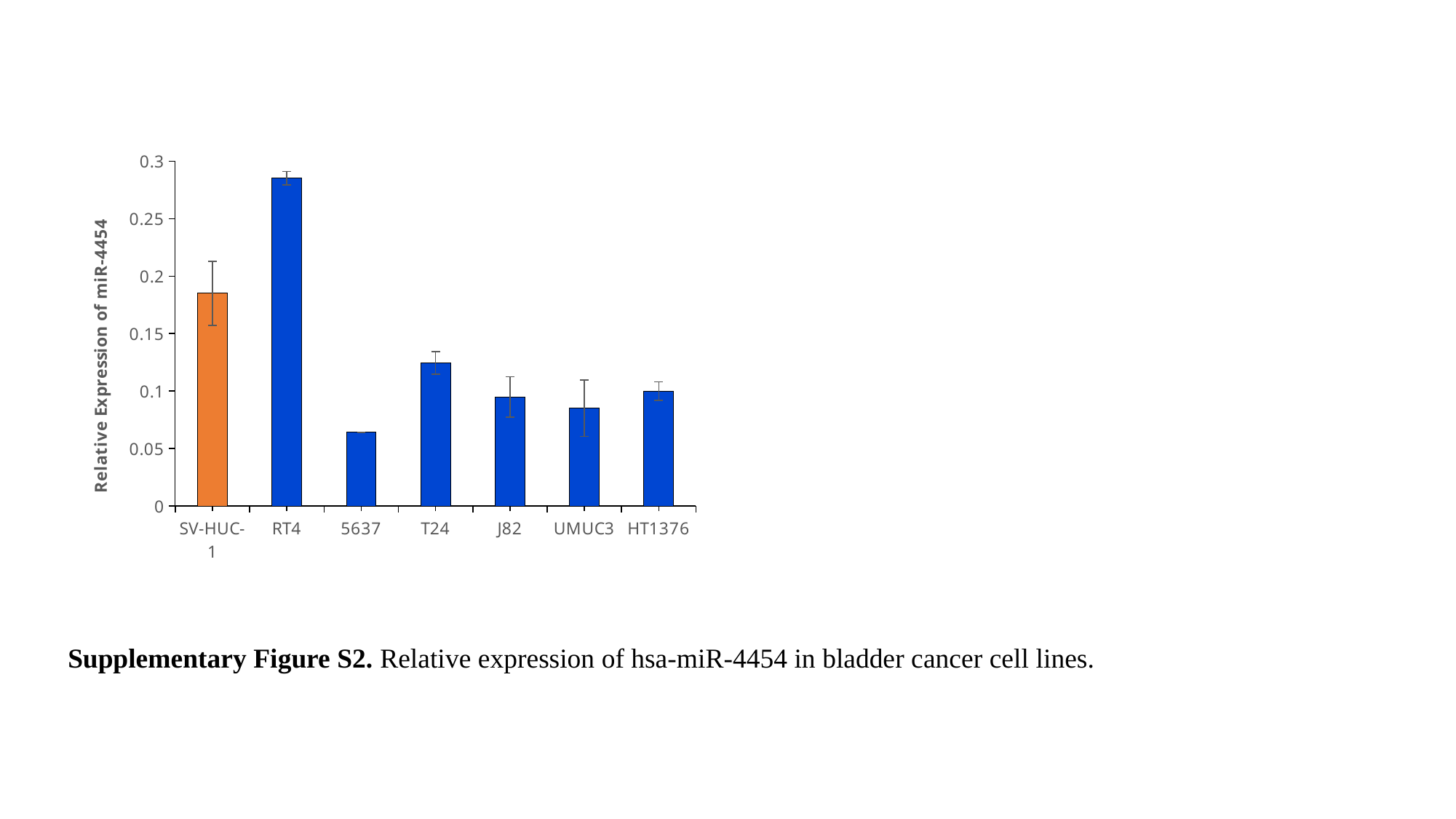

### Chart
| Category | |
|---|---|
| SV-HUC-1 | 0.18492140325439319 |
| RT4 | 0.28495074861059533 |
| 5637 | 0.06401722266080882 |
| T24 | 0.12441313185054652 |
| J82 | 0.09471157530806985 |
| UMUC3 | 0.08499869515747038 |
| HT1376 | 0.09988101972073547 |Supplementary Figure S2. Relative expression of hsa-miR-4454 in bladder cancer cell lines.
